# A Postmarket Surveillance Study on Electro-Neuro-Adaptive-Regulator Therapy

**DOI:** 10.1155/2014/341256

**Published:** 2014-06-05

**Authors:** Rod P. Bonello, Marc Cohen, John Reece, Arun Aggarwal, Curtis Rigney

**Affiliations:** ^1^School of Health Professions, Murdoch University, Perth 6150, Australia; ^2^School of Health Sciences, RMIT University, Melbourne 3000, Australia; ^3^School of Psychological Science, Australian College of Applied Psychology, Sydney 2000, Australia; ^4^University of Sydney, Sydney 2006, Australia; ^5^Macquarie University, North Ryde 2109, Australia

## Abstract

The Electro-Neuro-Adaptive-Regulator (ENAR) device is a hand-held electrotherapy which is applied using energetic medicine principles and aspects of acupuncture theory. The aim of this paper is to report the findings of a postmarket survey of persons who have used the ENAR device. The conditions for which the therapy was used and its perceived effectiveness are discussed. A web-based survey of Australian recipients of ENAR therapy was completed by 481 respondents. Most (76%) used ENAR exclusively for pain relief for musculoskeletal disorders, especially back, shoulder, and neck pain; 8% used ENAR exclusively for nonmusculoskeletal disorders; while 16% used ENAR for both. Respondents reported a mean reduction in pain of 70% (*t*(423) = 38.73, *P* < .001) and functional improvement of 62% (*t*(423) = 10.45, *P* < .001) using 11-point numerical rating scales. Following ENAR treatment, medication reduction was reported by 91% of respondents. Most respondents reported high satisfaction following ENAR therapy, with between 15 and 20% achieving complete pain relief. The self-delivery of ENAR may, in part, account for the high level of satisfaction.

## 1. Introduction 

The Electro-Neuro-Adaptive Regulator (ENAR) device is a hand-held, electrostimulator that is approved by the Australian Therapeutic Goods Administration (147761) as a Class IIa medical device and is designed for home use. The treatment may be self-administered or given by a medical practitioner or other therapist. The device is similar to a Transcutaneous Electrical Nerve Stimulator (TENS) device and is applied on the skin adjacent to an area of pain, or in a prescribed pattern which may incorporate acupuncture meridians. The main difference between TENS and ENAR is that ENAR incorporates a feedback system where its transmissions are constantly modified during application. The operating parameters of the ENAR device and a typical TENS device are contrasted in Table 1.

In terms of application, the ENAR device is used similarly to a hand-held TENS unit. Electrode pads can be adhered to the skin but, more usually, the device is drawn across the skin in fluid movements with light pressure. The device is commonly used for many of the same conditions as TENS, most usually for local pain relief.

The ENAR uses modulated electrical impulses that adjust electrical stimulation parameters based on feedback from the changing state of skin dose absorption [1]. These electrical impulses are purported to stimulate the dorsal root ganglion and trigger central neurotransmitter release that facilitates analgesia and better homeostasis.

As the ENAR sends electronic impulses via the skin to the brain, central pain mediating effects may also be occurring. The ENAR is believed to stimulate and measure the “reflex-biofeedback” changes in the skin by sensing areas of increased impedance called “asymmetry” or “key zones.” These areas of asymmetry are areas where normal homeostasis is not present. The ENAR reportedly causes a bioenergetic response that clears the neuroenergy pathways and restarts and regulates the flow of information to improve the natural flow of energy through the skin. Each impulse differs from the previous one, based on the changing state of the skin, its properties, electrically and chemically, and its hormone and fluid levels. It has been proposed that this allows ENAR to clear blockages and correct imbalances, so that normal healing and body functions can resume. It may be that the signals from the ENAR when applied to the body prioritise and amplify communication from the sensory nerve endings of the peripheral nervous system. This input is processed in the brain, which initiates a response transmitting the new information through the central nervous system to muscles and glands to coordinate and regulate responses. Slower acting chemical messengers, such as hormones and neuropeptides are also stimulated which affect more long term changes in physiological activities and result in pain relief.

The ENAR device has been available on the Australian market for over 10 years with more than 5000 devices in circulation. Very little research has been done on ENAR other than a small, randomised controlled trial on chronic neck pain sufferers by the lead author of this paper, which compared efficacy for ENAR and TENS over sham treatment for pain relief and improved function [2]. In that trial ENAR was used on the upper back and posterior neck. A statistically and clinically significant advantage was detected in ENAR treatment over both the TENS device and a sham treatment for pain reduction and functional improvement.

The objective of this paper is to report the findings of a postmarket survey of persons who have used the ENAR device for a variety of purposes. The conditions for which the therapy was used and its perceived effectiveness are discussed.

## 2. Methods

A postmarket survey was employed where all persons from the supplier's distribution database (over 4000 people) were contacted and asked to participate in the survey. A 33 question survey was designed to elicit information on experiences of using the device. The survey included a variety of questions using Likert options as well as open ended questions for qualitative assessment. Some questions catered for participants to enter multiple responses so that totals were not always limited to 100%. The survey was trialed by a handful of users as well as the author team prior to its distribution. Eligible participants were individuals who had used the device and were aged 18 years or older. The survey was open for a six-week period in mid-2013. Data were collected initially in Qualtrics and analysed using SPSS Version 21. The protocol was approved by the RMIT University Human Research Ethics Committee (ASEHAPP 09-13 COHEN).

## 3. Results

### 3.1. Respondent Characteristics

A total of 481 useable responses were received, giving a response rate of 12%. Respondents were aged 18 to 88 years (M = 54 years, SD = 14 years) with 69% being female. Respondents all had a history of chronic conditions (M = 6.4 years chronicity), for which they either acquired the ENAR device or sought treatment from an ENAR therapist.

### 3.2. Reasons for ENAR Use

Musculoskeletal complaints were identified as the primary problem by 405 (91.6%) respondents with the three most common conditions being (in order), back pain, shoulder pain, and neck pain. Thirty seven respondents (6%) had a nonmusculoskeletal problem as their “primary” complaint. Conditions with pain as a main symptom were categorised as “painful syndromes” and those with a primary symptom other than pain were categorised as “nonpainful syndromes.” A painful syndrome was reported by 88% of the respondents as their primary problem. Overall, 92% of respondents used ENAR for musculoskeletal problems and 24% for nonmusculoskeletal conditions.  Table 2 presents the profile of problems for which ENAR therapy was used.  Table 3 shows the breakdown between musculoskeletal and nonmusculoskeletal problems for which participants used ENAR.  Table 4 shows the proportion of painful conditions (painful syndrome) and nonpainful conditions (nonpainful syndrome) for which participants had used ENAR.

Although recognised in the market essentially as a device to treat pain, a substantial percentage of users (12%) employed ENAR to treat a range of nonpainful syndromes. Further, they reported very high levels of satisfaction for use of this device for such purposes. This finding is interesting and certainly worthy of further investigation in future studies.

### 3.3. Who Diagnosed These Respondents?

When asking respondents what their health problems are, it is important to determine if these problems were diagnosed by a professional or if they were self-diagnosed. Where a high percentage of diagnoses are made by a person other than a health professional this may cast doubt on the veracity of the actual diagnosis. In this study 30% of respondents had self-diagnosed their problem; 65% of the diagnoses were medical, and the remainder was made by CAM therapists. (CAM therapists included persons registered under Australian Health Practitioner Regulation Agency as well as nonregistered therapists such as naturopaths, herbalists, homoeopaths, and masseurs).

### 3.4. Chronicity before Commencing ENAR Therapy

For the purpose of this paper, a condition was regarded as “chronic” if it had been present for not less than three months. The ENAR device was used most commonly for chronic problems with more than half (55%) of the respondents stating they had experienced their primary problem for “years” (rather than “days” or “months”) with an average chronicity of 6.4 years across the whole sample.

### 3.5. Number and Duration of ENAR Treatments

Respondents showed a bipolar distribution with respect to number of treatments they had. They either followed a short term treatment protocol (1–4 sessions) or a lengthy protocol of more than 20 sessions (M across the whole sample = 5.6 months; SD = 1.24 months). The average therapy session lasted 30.01 minutes (SD = 18.0 minutes).

### 3.6. Changes in Pain Level Associated with ENAR Therapy

**Figure 1:**
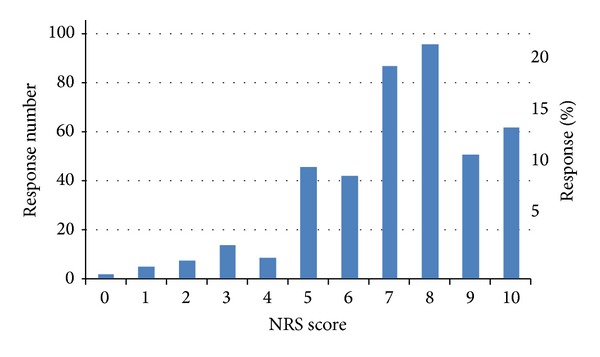
Frequency of NRS pain levels prior to ENAR therapy (Mean = 7.16).

**Figure 2:**
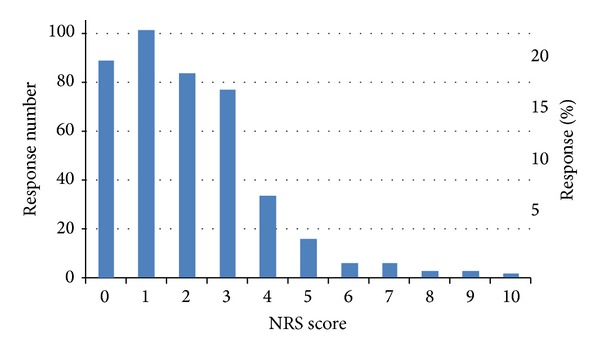
Frequency of NRS pain level after ENAR therapy (Mean = 2.04).

Using an 11-point pain numerical rating scale (NRS), there was a statistically significant reduction in self-reported pain after ENAR treatment, with mean pain scores reducing from 7.16 before treatment to 2.04 after treatment, *t*(423) = 38.73, *P* < .001. The percentage change in pain rating for each participant was calculated, revealing a mean percentage reduction of pain of 66.2%, which is likely to be of notable clinical significance as a change in pain NRS of at least 1.3 scale points (i.e., about 13%) is reported to represent a clinically beneficial difference [3, 4]. Forty-one percent of respondents stated that the effects of ENAR treatment lasted for some days, 28% found that the effects lasted for months, and 18% reported pain reduction that lasted years. Figures 1 and 2 show NRS pain scores before and after ENAR therapy, respectively.

### 3.7. Changes in Functional Activity Associated with ENAR Therapy

There was a statistically significant improvement in NRS self-reported functional activity level after ENAR treatment with mean functional activity ratings being 3.49 before and 5.65 (out of 10) after ENAR treatment, *t*(423) = 10.45, *P* < .001. The percentage improvement in functional activity was calculated for each individual, revealing mean improvement in functional activity of 78.9%; this is likely to be clinically relevant as it well exceeds a change in 2.0 functional NRS points (i.e., about 20%), which, in line with NRS pain changes, can be notionally regarded as a clinically significant therapeutic goal. There was an observed correlation between age and improvement in activity, with older participants demonstrating greater improvement, *r* = .16, *P* = .001; however, pain reduction was not related to age, *r* = − .07, *P* = .14.

There was no significant relationship between chronicity of complaint and pain reduction, *r* = −.07, *P* = .08, but there was a significant positive correlation between chronicity of complaint and improvement in activity, *r* = .10, *P* = .009. Thirty-five percent of respondents reported that the effects of ENAR on functional improvement lasted for some days. Almost as many (33%) found that the effects lasted for months. A substantial proportion (23%) claimed a treatment effect that lasted years, despite this being a population of chronic sufferers.

### 3.8. Effects of ENAR Therapy on Medication Use

Over half of the respondents (54.5%) were taking medication prior to commencing ENAR therapy for their primary problem. Of these, 91.2% reported that because of ENAR treatment they were able to reduce or eliminate medication use for their primary problem. In this group, 42% were able to cease medication altogether. Only one respondent of the 206 who reported a change in medication following ENAR therapy reported an increased use of medication.

### 3.9. Perceptions of End-Users on the Overall Effectiveness of ENAR Therapy

Respondents reported ENAR to be a highly successful therapy. ENAR was found by almost all respondents (97.8%) to have a positive effect on their primary problem. Almost two thirds (63.0%) reported “great effectiveness” and 19.3% claimed that ENAR “cured” their problem.  Table 5 reviews these claims of cure by showing specific conditions treated by ENAR and the percentage of respondents who said that ENAR cured their condition—giving a notional “cure rate.”

### 3.10. Respondent Perceptions of Comparative Effectiveness

**Figure 3:**
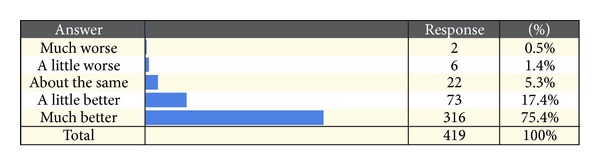
Perception of the effectiveness of ENAR compared to other therapies previously tried.

Respondents were asked to rate the effectiveness of ENAR compared with other therapies that they had tried previously. This was a broad question that did not consider the type of other therapies that participants may have chosen. On a five-point scale ranging from “Much worse” to “Much better,” 75.4% of respondents rated ENAR as much better. In total, 92.8% rated ENAR as either “A little better” or “Much better.” Only 1.9% rated ENAR as either “A little worse” or “Much worse” than other alternative therapies they had tried. Sixty-two respondents chose not to answer this question.  Figure 3 shows the relative effectiveness of ENAR compared to other therapies as reported by respondents.

## 4. Discussion

Although recognised in the market essentially as a device to treat pain, many users employ ENAR to treat a range of nonpainful syndromes. Further, they report very high levels of satisfaction for use of the device for such purposes. Most ENAR users are enthusiastic uptakers of the therapy with a large proportion of respondents being home users.

Older users of ENAR reported better improvement in functional activity than younger patients. This may be accounted for by the fact that older persons carry age-related functional impairments and would therefore have had more scope for improvement in this regard than younger respondents. The human body changes dramatically with age in terms of structural integrity (e.g., osteoporosis and joint degeneration), organ function (e.g., liver and brain degeneration), as well as emotional responses [5, 6]. It should be noted, however, that the correlation in support of this finding, although significant, was weak; hence, the clinical meaningfulness of this finding is questionable.

In examining the impact and value of any new or alternate therapy, an important consideration is the ability of patients who use that therapy to become less reliant on other forms of treatment. In the area of pain relief, some medications are a common source of harmful side-effects. In addition, prescription drugs that are heavily subsidised are a major drain on the National health care budget. The results of this survey support the notion that ENAR treatment appears to offer an alternative to drug therapy in some cases.

Although tempting to regard ENAR as an analgesic or anti-inflammatory, it appears that it has other effects that may account for the considerable effectiveness reported for nonpain based conditions. At least part of this may be due to a placebo effect and the fact that as a patient-directed therapy, the ENAR device provides patients with a degree of empowerment over their condition. Features of self-directed therapy have been investigated in the area of cognitive behavioural therapy, where studies commonly show that such approaches are either better than or at least as good as therapist directed treatments [7]. In part, such findings may point to a degree of consumer satisfaction which naturally accompanies the purchase of a product or service, independently of its ultimate value. The quality of the therapeutic environment has been investigated with respect to patients having personal control over their therapy in a hospital [8]. It was found that higher levels of personal control were associated with enhanced therapeutic potential. Similarly, in a placebo controlled experimental study, Geers and coworkers found that treatment effectiveness was enhanced when participants were given control over choice of therapy [9]. While these factors appear to be present, they probably do not account for the strong trends shown in the data set. More rigorous investigation of the effects of ENAR under controlled trial conditions is desirable.

The sample represents an age and gender profile consistent with the results from a National Institutes of Health (USA) study that found that women aged between 46 and 64 years were the most likely sector to use CAM therapies [10]. Similarly, in Australia, a 2007 survey found that CAM use was more common among females, yet in a lower (18–35 years) age bracket [11]. Women comprised almost 70% of the sample and are known to seek CAM therapy for a wider range of problems than do men [12].

The data from this survey provides tentative evidence that ENAR might be an effective treatment for many painful musculoskeletal complaints although a wide range of other types of problems was also reported to have been successfully managed. Most respondents reported high satisfaction and a reduction in medication use following ENAR therapy, with between 15 and 20% achieving complete pain relief.

Claims of cure must be treated with some caution. Disorders have a natural history which eventually includes the cessation of symptoms independently of any treatment. Many conditions are episodic and a remission period may be mistaken as permanent eradication of the condition. In addition, placebo effects may make a condition appear to have ceased. Notwithstanding this, the responses showing cure were found to be relatively consistent for most conditions, with the so-called cure rate in the 15–20% range. The exception was skin conditions, with a cure rate of 26%. Additional caution must be taken with the entry for stroke rehabilitation owing to the small number of respondents; however, it is noted that its “cure” rate falls within the same range as that of the other conditions.

## 5. Conclusion

The self-use nature of the ENAR device is in keeping with neoliberal attitudes that embrace higher levels of personal control or empowerment over one's health. This may, in part, account for the high level of satisfaction of respondents with ENAR therapy, especially when contrasted to their use of other therapies. We would contend that the results from this study are enough to recommend a more rigorous clinical trial to evaluate the effectiveness of ENAR therapy under controlled conditions.

This study has found that patients who have used the ENAR device report very high levels of satisfaction with the therapy. They reported that the therapy was associated with reduction in pain and improvement in function. They reported reduction in their use of medications and that they preferred ENAR to other treatments they had tried previously. Although in some ways similar to TENS, based on these results, it appears that ENAR gives patients a far better experience than TENS. This study did not investigate the physiological basis for such differences. Such an investigation needs to take place to better understand the potential applications for ENAR.

## Figures and Tables

**Table 1:** Operating parameters ENAR compared to a typical hand-held TENS device.

	ENAR	TENS
Power supply	9 V battery	9 V battery
Operating or pulse frequency	14–320 Hz	2–150 Hz
Sweep frequency	10–120 Hz	20–80 Hz
Sweep period	5–9 seconds	6–10 seconds
Pulse duration	105–250 *μ*s ±5%	100–200 *μ*s

**Table 2:** Primary problem for which ENAR was used.

Summary of responses		
Region or system involved	Count	%
Low back*	111	25
Shoulder*	60	14
Neck*	56	13
Knee*	42	10
Neurological	31	7
Ankle/foot*	27	6
Arthritis*	25	6
Fibromyalgia*	25	6
Hip*	22	5
Headache	16	4
Wrist/hand*	14	3
Thoracic spine*	14	3
Digestive	13	3
Other Head	12	3
Emotional	9	2
Elbow*	9	2
Skin	8	2
Hormonal	6	1
Cardiovascular	6	1
Genitourinary	3	1
General health	2	0

Notes: a number of respondents cited more than one problem as the reason they commenced ENAR therapy. Musculoskeletal conditions are shown by ∗.

**Table 3:** Whether or not the primary problem was a musculoskeletal complaint.

Type of problem	Count	%
Musculoskeletal (MSK) syndrome only	336	76
Nonmusculoskeletal syndrome only	37	8
Used for both MSK and Non-MSK syndrome	69	16

Total	442	100%

**Table 4:** Whether or not the primary problem was a pain syndrome.

Region or system involved	Count	%
Painful syndrome	387	88
Nonpainful syndrome	55	12

Total	442	100%

**Table 5:** “Cure” rates for five named conditions and two condition groups.

Condition	Total respondents	Number of reported “cure”	% cure rate
Spinal or back problems	304	45	14.8%
Neurological problems	107	20	18.7%
Stroke rehabilitation	16	3	18.8%
Digestion or food sensitivity	94	18	19.1%
Skin or cosmetic problems	87	23	26.4%

Musculoskeletal conditions	327	54	16.5%
Pain syndromes	353	61	17.3%

## Limitations to This Study

This was a retrospective survey, and recall bias may have resulted in participants not accurately recollecting details of their condition. This study did not have a placebo control group; hence, the impact of a range of threats to internal validity cannot be accounted for. Because of this, it is not possible to measure the magnitude of efficacy of ENAR therapy beyond single-group pre- and posttest comparisons. Therefore, the conclusions made in this report require confirmation in more rigorous studies. Finally, the results relied solely on self-report, with no corroborating evidence from health care professionals.
